# Is job strain associated with a higher risk of type 2 diabetes mellitus? A systematic review and meta-analysis of prospective cohort studies

**DOI:** 10.5271/sjweh.3938

**Published:** 2021-04-27

**Authors:** Wenzhen Li, Guilin Yi, Zhenlong Chen, Xiayun Dai, Jie Wu, Ying Peng, Wenyu Ruan, Zuxun Lu, Dongming Wang

**Affiliations:** Department of Social Medicine and Health Management, School of Public Health, Tongji Medical College, Huazhong University of Science and Technology, Wuhan, Hubei, People’s Republic of China; Wuhan Prevention and Treatment Center for Occupational Diseases, Wuhan, Hubei, People’s Republic of China; Wuhan Centers for Disease Prevention and Control, Wuhan, Hubei, People’s Republic of China; Shangluo Central Hospital, Shangluo, Shanxi, People’s Republic of China; Department of Occupational & Environmental Health, School of Public Health, Tongji Medical College, Huazhong University of Science and Technology, Wuhan, Hubei, People’s Republic of China; Key Laboratory of Environment and Health, Ministry of Education & Ministry of Environmental Protection, and State Key Laboratory of Environmental Health (Incubating), School of Public Health, Tongji Medical College, Huazhong University of Science and Technology, Wuhan, Hubei, People’s Republic of China

**Keywords:** job control, job demand, psychosocial, work stress

## Abstract

**Objectives:**

Epidemiological studies have explored the relationship between work-related stress and the risk of type 2 diabetes mellitus (T2DM), but it remains unclear on whether work-related stress could increase the risk of T2DM. We aimed to evaluate the association between job strain and the risk of T2DM.

**Methods:**

We searched PubMed and Web of Science up to April 2019. Summary risk estimates were calculated by random-effect models. And the analysis was also conducted stratifying by gender, study location, smoking, drinking, body mass index, physical activity, family history of T2DM, education and T2DM ascertainment. Studies with binary job strain and quadrants based on the job strain model were analyzed separately.

**Results:**

A total of nine studies with 210 939 participants free of T2DM were included in this analysis. High job strain (high job demands and low control) was associated with the overall risk of T2DM compared with no job strain (all other combinations) [relative risk (RR) 1.16, 95% confidence interval (CI) 1.03–1.31], and the association was more evident in women (RR 1.48, 95% CI 1.02–2.14). A statistically significant association was also observed when using high strain as a category (job strain quadrants) rather than binary variable (RR 1.62, 95% CI 1.04–2.55) in women but not men.

**Conclusions:**

Our study suggests that job strain is an important risk factor for T2DM, especially among women. Appropriate preventive interventions in populations with high job strain would contribute to a reduction in T2DM risk.

Recently, studies have shown that work-related stress, a known occupational hazard, might increase the risk of cardiovascular disease (CVD), cancer and death (1–4). Psychosocial factors at work played a pivotal role in the pathogenesis and progression of CVD and cancer, involving activation of sympathetic nervous system and dysregulation of the hypothalamic–pituitary–­adrenal axis, which could accelerate the development of the metabolic syndrome, increase the production of cortisol, trigger and maintain chronic inflammation, and lead to dysrhythmia (5–8).

Type 2 diabetes mellitus (T2DM), one of the most prevalent chronic diseases, is considered to be one of the major public health challenges in both developed and developing countries (9). Work-related psychosocial stress has been hypothesized to increase the individual risk of T2DM; however, the results of the studies examining the association between work-related stress and T2DM risk are inconsistent. In 2012, a meta-analysis conducted by Cosgrove et al (10) showed that high psychosocial stress was not directly associated with increased risk of T2DM, the significant heterogeneity in the design of the original studies and the work-related stress models may introduce bias into the meta-analysis. Another meta-analysis published in 2016 (11) suggested that no significant association was found between work-related stress and risk of T2DM based on seven prospective cohort studies. Notably, the psychosocial work characteristics and the reference of job strain between included studies were different, for example, Smith et al’s study (12) presented the hazard ratios (HR) associated with separate dimensions of psychosocial work environment and T2DM, while Nyberg et al’s study (13) presented the HR for job strain (high demands and low control) compared with no job strain (all other combinations) based on job strain model (14). Moreover, the literature search was limited to September 2014, and some new, high quality studies have been reported in subsequent several years.

We therefore carried out an updated systematic review and meta-analysis of prospective cohort studies to comprehensively explore the relationship between job strain and T2DM risk. Job strain was measured with sets of questions from the validated Job Content Questionnaire and Demand–Control Questionnaire in these prospective cohort studies (15), and the scores were assessed according to Karasek and colleague’s job strain model (16).

## Methods

### Search strategy

This systematic review was conducted in accordance with the Meta-analysis of Observational Studies in Epidemiology (MOOSE) guidelines (17). Eligible studies were identified from review articles, computer-aided literature searches in the PubMed and Web of Science, using combinations of the search terms: (‘work stress’ or ‘job strain’ or ‘job stress’ or ‘occupational stress’ or ‘work-related psychosocial stress’) and (‘diabetes mellitus’ or ‘type 2 diabetes mellitus’ or ‘diabetes’), up to 3 April 2019. The search was restricted to human studies. No restrictions were imposed on the language of publications. Abstracts, non-original papers (reviews, editorials, or letters), grey literature, and unpublished results or information were not included. We also reviewed the reference lists of all included studies and relevant reviews.

### Inclusion criteria

A study was included in the meta-analysis if it: (i) was a cohort design, (ii) evaluated the association between job strain and the T2DM risk, (iii) reported estimates of relative risk (RR) or HR with 95% confidence intervals (CI), and (iv) defined the job strain according to the Job Content Questionnaire (JCQ) or derivatives of the JCQ, and scores of the validated JCQ based on the job strain model. Only the most recent and informative studies were included, when multiple reports were published on the same population.

### Data collection

Two investigators extracted detailed information in the predefined criteria to ensure consistency in data collection independently. Any disagreements were discussed to obtain consistency by another investigator. We extracted the following data from the studies included in the final analyses: name of first author, year of publication, country, characteristics of study population at baseline, duration of follow-up, number of cases, number of participants, risk estimates and corresponding 95% CI, and covariates adjusted in the statistical analysis. Any article stratified by gender or age was treated as two separate reports.

### Quality assessment

Two reviewers independently performed the quality assessment using the Newcastle-Ottawa Scale (18), which is a 9-point scale allocating points based on the selection process of cohorts (0–4 points), the comparability of cohorts (0–2 points) and the identification of the exposure and the outcomes of study participants (0–3 points). We considered a study with a score of ≥6 as a high-quality study.

### Statistical analysis

We pooled multivariable adjusted risk estimates when such estimates were reported. If adjusted analysis was unavailable, we used the unadjusted estimate. In addition, the HR were regarded directly as RR in our analysis.

Binary job strain was defined as job strain (high demands and low control) versus no strain (all other categories combined), and/or job strain categories, or quadrants based on the job strain model, including high strain job (high demands and low control), active job (high demands and high control), passive job (low demands and low control), and low strain job (low demands and high control). Job strain was modeled both as a binary exposure (strain versus no strain) and as a categorical exposure (high strain, active job, and passive job versus low strain). We used the study-specific RR for job strain versus no strain, or high strain, active job, and passive job versus low strain.

We calculated summary estimates of the RR/HR using random-effects models, which considered both within- and between-study variation. Heterogeneity of effect size across studies was tested by I^2^ statistics, the following cut-off points were used: <30% (little or no heterogeneity), 30–75% (moderate heterogeneity), and >75% (high heterogeneity) (19). Sources of heterogeneity were investigated by meta-regression analyses and subgroup analyses. Analyses were separated based on sex, study location and whether the results were adjusted by smoking, drinking, body mass index (BMI), physical, family history of T2DM, education and T2DM ascertainment. Visual inspection of funnel plots, Begg’s and Egger’s tests were used to evaluate the publication bias (20, 21). The meta-analysis was performed using STATA version 12.0 (Stata Corp, College Station, TX, USA). All P values are two tailed, and we set P<0.05 as the threshold for significance.

## Results

### Identification of relevant studies

**Figure 1:**
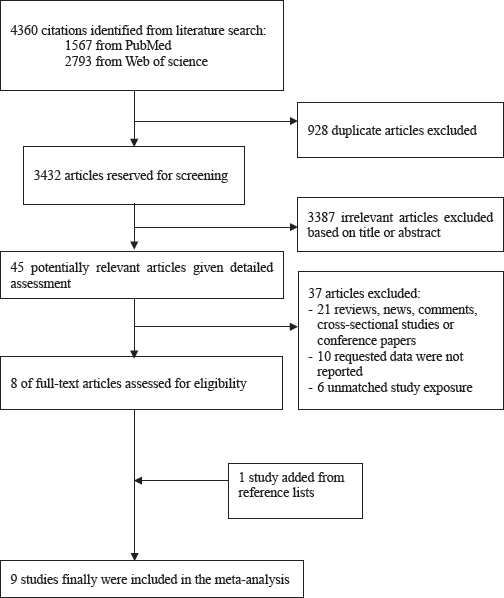
Flow chart for selection of eligible studies.

Detailed process of the study selection is described in figure 1. Briefly, from the initial searched literatures, we identified and screened articles (PubMed: N=1567, Web of Science: N=2793). Of which, the majority were excluded because they were reviews, animal studies, not relevant to our analysis or association of interest was not evaluated, or requested data were not reported. By examining the full texts of eight articles, we added one study from reference lists. Finally, nine studies were included in our meta-analysis.

### Description of studies included in the final analysis

We identified nine studies on job strain and T2DM risk and the details of included studies are presented in table 1. The selected studies were published between 1999–2017. The study samples ranged from 584–124 808, with a total of 210 939, and the number of cases of T2DM ranged from 34–3703, with a total of 5105. The duration of follow-up for incident T2DM ranged from 5.8–12.7 years. One cohort was conducted in Japan (22), three in Sweden (23–25), one in UK (26), one in Europe (13), two in US (27, 28) and one in Germany (29). In total, one study reported result for women only (27), one study reported result for men only (22), and one study reported result for both men and women combined only (29). Four studies (13, 22, 25, 26) presented RR for job strain versus no strain, five studies (23, 24, 27–29) reported RR for high strain, active job, and passive job versus low strain, respectively. The average score for included studies was 7.8 (range 7–9), supplementary material (www.sjweh.fi/show_abstract.php?abstract_id=3938), table S1.

**Table 1:** Main characteristics of cohort studies included in the meta-analysis. [M=men; W=women; BMI=body mass index.]

Study	Country	Age (years)	Gender	Follow-up (years)	Partici-pants	Cases	Exposure definition & comparison	Case ascertainment	Adjustment for covariates	Study quality
Mutambudzi & Javed, 2016 (28)	US	≥50	M (43%) / W	7	1396	167	High strain, active job, and passive job vs low strain	Self-reported	BMI, physical activity, education, race, gender, alcohol use, average work hours/week, occupational category, marital status, insurance coverage, and hypertension	7
Huth et al, 2014 (29)	Germany	29-66	M (62.8%) / W	12.7	5337	291	High strain, active job, and passive job vs low strain	Self-reported and the date of diagnosis were validated by hospital records or physicians.	Sex, age, baseline survey, education, physical intensity of work, parental history of diabetes, living alone, physical inactivity, smoking, alcohol intake, BMI	9
Nyberg et al, 2014 (13)	European	44.1 (mean)	M (43.27%) / W	10.3	124 808	3703	High job demands and low control vs others	National health registers, clinical screening, and self-reports	Age, sex, and socioeconomic status	7
Kawakami et al, 1999 (22)	Japan	≥18	M	8	2194	34	High job demands and low control vs others	Medical check up	None	7
Heraclides et al, 2009 (26)	UK	35–55	M (70.67%) / W	11.6	5895	308	High job demands and low control vs others	Glucose tolerance test	Age	8
Kroenke et al, 2006 (27)	US	29–46	W	69.3 months	62 574	86	High strain, active job, and passive job vs low strain	Questionnaire and verified through medical records	Age ,BMI, family history of diabetes , work hours , rotating night-shift work , hours at work sitting, , job support, hours per week of work at home, leisure-time physical activity, smoking, alcohol intake, transunsaturated fat intake , glycemic load , caffeine intake , marital status, number of children, menopausal status , vitamin supplementation, and aspirin use	8
Norberg et al, 2007 (23)	Sweden	40-60	M (58.9%) / W	7.8	584	191	High strain, active job, and passive job vs low strain	Diagnosis registers	Age, sex and year of health survey	8
Eriksson et al, 2013 (24)	Sweden	35–56	M (41%) / W	8–10	5432	171	High strain, active job, and passive job vs low strain	Questionnaire and glucose tolerance test	Age, education, BMI, physical activity, smoking, family history of diabetes, and psychological distress	9
Pan et al, 2017 (25)	Sweden	≥60	M (35.4%) / W	6	2719	154	High job demands and low control vs others	Glycated haemoglobin level, self-report, hypoglycaemic medication use and clinical records	Sex, age, education level, vital status, follow-up time, BMI, smoking, alcohol consumption and physical activity, history of vascular diseases and the history of heart operation, cholesterol, blood pressure and depression	7

### Association of high job strain with T2DM risk

**Figure 2:**
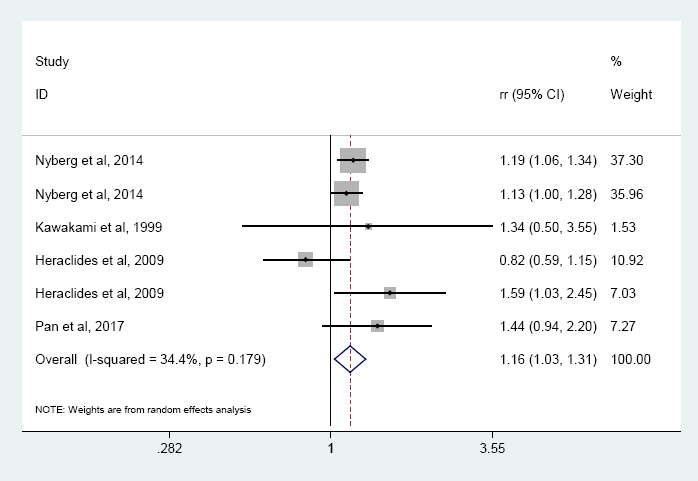
The association of high job strain with T2DM risk.

Four prospective studies (13, 22, 25, 26) with six reports were included in the high job strain meta-analysis. Results indicated that high job strain (high job demands and low control) was associated with the overall risk of T2DM, compared with no job strain (all other combinations) (RR 1.16, 95% CI 1.03–1.31, I^2^=34.4%, P=0.179) (figure 2). Especially, high job strain was significantly associated with the risk of T2DM among women (RR 1.48, 95% CI 1.02–2.14, I^2^=61.3%, P=0.051) with moderate heterogeneity and both gender group (RR 1.15, 95% CI 1.06–2.14, I^2^=0.00%, P=0.437) without heterogeneity in the subgroup analysis, but not for men. In addition, the association was observed in Europe but not Asia (table 2).

**Table 2:** Subgroup analysis of relative risk of high job strain with T2DM risk. [T2DM=type 2 diabetes mellitus; BMI=body mass index; RR=relative risk; CI=confidence interval.]

	Reports (N)	RR	95% CI	I^2^ (%)	P ^a^

Gender ^b^					
Men	5	1.02	0.76–1.36	43.5	0.132
Women	4	1.48	1.02–2.14	61.3	0.057
Study location					
Europe	5	1.16	1.01–1.33	46.9	0.110
Asia	1	1.34	0.50–3.57		
T2DM ascertainment					
Objectively defined	6	1.16	1.03–1.31	34.4	0.179
Self-reported	0				
Controlling smoking in models					
Yes	1	1.14	0.94–2.20		
No	5	1.14	1.00–1.30	39.3	0.159
Controlling drinking in models					
Yes	1	1.14	0.94–2.20		
No	5	1.14	1.00–1.30	39.3	0.159
Controlling physical activity in models					
Yes	1	1.14	0.94–2.20		
No	5	1.14	1.00–1.30	39.3	0.159
Controlling BMI in models					
Yes	1	1.14	0.94–2.20		
No	5	1.14	1.00–1.30	39.3	0.159
Controlling family history of DM in models					
Yes	1	1.14	0.94–2.20		
No	5	1.14	1.00–1.30	39.3	0.159
Controlling education in models					
Yes	1	1.14	0.94–2.20		
No	5	1.14	1.00–1.30	39.3	0.159

a P-value for homogeneity.

b Pan et al (2017) stratified by gender and age (60 years old population and population aged ≥ 66 years old) were treated as for four separate reports.

### Association between job strain model quadrants and T2DM risk

**Figure 3:**
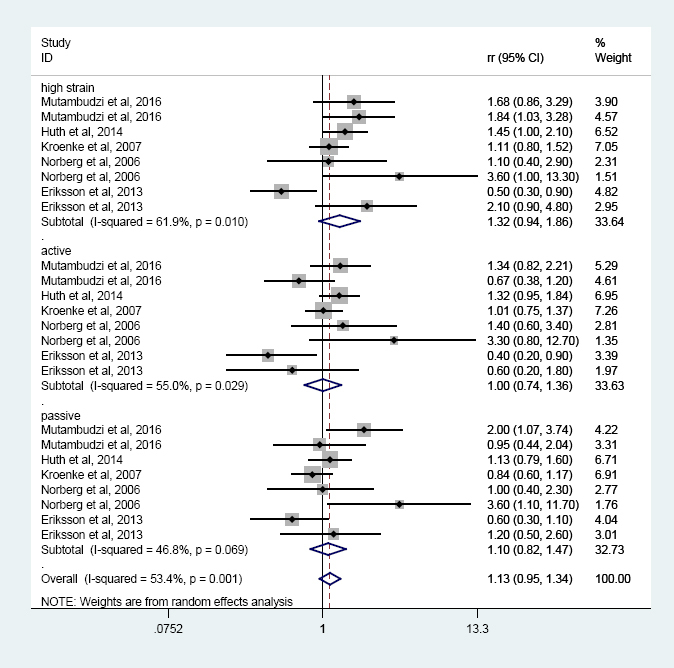
The association between job strain model quadrants and T2DM risk.

Five prospective studies (23, 24, 27–29) were included in the job strain model quadrants meta-analysis. No significant association was found between the quadrants of the job strain model (versus low strain) and T2DM risk, and similar results were found in the three categories of high strain, active job, and passive job when compared with low strain (figure 3). However, the subgroup analysis indicated that the T2DM risk increased 62% among women in high strain category with moderate heterogeneity (RR 1.62, 95% CI 1.04–2.55, I^2^=48.2%, P=0.122), but not for men (table 3).

**Table 3:** Subgroup analysis of job strain model quadrants and T2DM risk. [T2DM=type 2 diabetes mellitus; BMI=body mass index; RR=relative risk; CI=confidence interval].

	Reports (N)	RR	95 %CI	I^2^ (%)	P ^a^

Gender					
Men	9	0.98	0.66–1.44	66.4	0.003
High strain	3	0.95	0.42–2.14	74.4	0.020
Active	3	0.92	0.42–2.02	73.8	0.022
Passive	3	1.07	0.50–2.29	71	0.003
Women	12	1.18	0.92–1.52	49.6	0.026
High strain	4	1.62	1.04–2.55	48.2	0.122
Active	4	0.93	0.59–1.46	44	0.147
Passive	4	1.12	0.69–1.83	47.3	0.127
Both	3	1.11	0.90–1.37	61.6	0.008
High strain	1	1.45	1.00–2.10		
Active	1	1.32	0.95–1.84		
Passive	1	1.13	0.79–1.60		
Study location					
North American	9	1.14	0.92–1.41	44.8	0.070
High strain	3	1.38	0.98–1.94	30.4	0.238
Active	3	1.00	0.72–1.37	37.6	0.201
Passive	3	1.13	0.65–1.97	65.4	0.056
Europe	15	1.12	0.85–1.48	59.8	0.002
High strain	5	1.29	0.70–2.41	73.8	0.004
Active	5	1.01	0.55–1.87	66.6	0.017
Passive	5	1.09	0.72–1.67	45.1	0.122
T2DM ascertainment					
Self-reported	6				
High strain	2	1.77	1.14–2.74	0	0.841
Active	2	0.96	0.49–1.90	68.8	0.074
Passive	2	1.43	0.69–2.95	54.0	0.140
Objectively defined	18				
High strain	6	1.21	0.79–1.85	67.5	0.009
Active	6	1.01	0.68–1.50	59.2	0.032
Passive	6	1.00	0.73–1.37	41.3	0.130
Controlling smoking in models					
Yes	18	1.06	0.89–1.27	56.9	0.002
High strain	6	1.27	0.88–1.83	68.2	0.008
Active	6	0.92	0.67–1.27	59.7	0.030
Passive	6	1.03	0.77–0.37	43.2	0.117
No	6	1.73	1.08–2.78	17.6	0.300
High strain	2	1.84	0.58–5.84	50.8	0.154
Active	2	1.80	0.84–3.88	5.7	0.303
Passive	2	1.78	0.51–3.20	65.7	0.088
Controlling drinking in models					
Yes	12	1.18	1.01–1.38	35.6	0.106
High strain	4	1.36	1.09–1.68	2.50	0.380
Active	4	1.09	0.84–1.41	39.3	0.176
Passive	4	1.11	0.79–1.55	50.2	0.111
No	12	1.10	0.72–1.66	62.8	0.002
High strain	4	1.30	0.53–3.21	76.7	0.005
Active	4	0.93	0.39–2.22	67.3	0.027
Passive	4	1.13	0.59–2.18	57.6	0.070
Controlling physical activity in models					
Yes	12	0.97	0.79–1.20	53.6	0.005
High strain	4	1.10	0.68–1.78	75.6	0.006
Active	4	0.87	0.56–1.36	67.3	0.027
Passive	4	0.92	0.72–1.19	17.7	0.303
No	12	1.44	1.10–1.90	33.3	0.124
High strain	4	1.75	1.20–2.57	0	0.556
Active	4	1.20	0.71–2.04	51.4	0.104
Passive	4	1.52	0.89–2.61	41.2	0.164
Controlling BMI in models					
Yes	18	1.06	0.89–1.27	56.9	0.002
High strain	6	1.27	0.88–1.83	68.2	0.008
Active	6	0.92	0.67–1.27	59.7	0.030
Passive	6	1.03	0.77–0.37	43.2	0.117
No	6	1.73	1.08–2.78	17.6	0.300
High strain	2	1.84	0.58–5.84	50.8	0.154
Active	2	1.80	0.84–3.88	5.7	0.303
Passive	2	1.78	0.51–3.20	65.7	0.088
Controlling history of T2DM in models					
Yes	12	0.97	0.79–1.20	53.6	0.005
High strain	4	1.10	0.68–1.78	75.6	0.006
Active	4	0.87	0.56–1.36	67.3	0.027
Passive	4	0.92	0.72–1.19	17.7	0.303
No	12	1.44	1.10–1.90	33.3	0.124
High strain	4	1.75	1.20–2.57	0	0.556
Active	4	1.20	0.71–2.04	51.4	0.104
Passive	4	1.52	0.89–2.61	41.2	0.164
Controlling education in models					
Yes	15				
High strain	5	1.33	0.81–2.19	73.6	0.004
Active	5	0.86	0.54–1.35	67.7	0.015
Passive	5	1.10	0.77–1.58	43.2	1.133
No	9				
High strain	3	1.31	0.76–2.25	33.6	0.222
Active	3	1.26	0.75–2.09	34.0	0.220
Passive	3	1.22	0.59–2.52	63.0	0.067

a P-value for heterogeneity

### Subgroup and sensitivity analyses

Subgroup analysis was conducted to test the stability of the results. Results changed when controlling for smoking, drinking, BMI, alcohol consumption, physical activity family history of T2DM, education and T2DM ascertainment in both the subgroup analyses of the association between high job strain and T2DM risk (table 2) and with respect to the association between job strain model quadrants and T2DM risk (table 3). In addition, sensitivity analysis excluding a single study in turn did not alter the combined RR (supplementary figures S1a and S1b).

### Publication bias

Visual inspection of funnel plots failed to identify substantial asymmetry [supplementary figures S2(A) and S2(B)]. There was no evidence of publication bias among the studies was found by Begg’s rank correlation test and Egger’s linear regression test for high job strain (Begg’s test P=0.602; Egger’s P=0.612) and job strain model quadrants (Begg’s test P=0.189; Egger’s P=0.418).

## Discussion

Our meta-analysis suggested high job strain (high job demands and low control) increased overall risk of T2DM when compared with no job strain (all other combinations). And the result was significant among women but not men. Studies have showed that women tend to work more in high stress occupations such as healthcare jobs and education (30). Another explanation could be that women had lower degree of freedom at work, a higher stress load due to non-paid work compared to men, and experienced the gender inequality during work life (31). Women may therefore be at higher risk of experiencing adverse health-related outcomes such as T2DM due to the higher proportion experiencing the job to be high-strain. Besides, combined high strain, active job, and passive job were not associated with T2DM risk when job strain was divided into four categories according to job strain model; however, the T2DM risk increased by 62% among women in the high strain category.

Our study was inconsistent with two previous meta-analyses (10, 11), both of which indicated that job strain is not directly associated with increased risk of T2DM. However, Hua Sui et al’s study (11) showed that the highest group of job strain was associated with T2DM risk among women (RR 1.22, 95% CI 1.01–1.46), when compared with the lowest category. Our results found the risk to be greater (RR 1.62, 95% CI 1.04–2.55). Furthermore, the subgroup analyses showed that results changed when controlling for smoking, drinking, BMI, alcohol consumption, physical activity, family history of T2DM, education and T2DM ascertainment, which suggested that lifestyle factors may play a key role in the relationship between job strain and T2DM risk. Stressed individuals are more likely to smoke, increase alcohol consumption, and be obese than stress-free individuals (32–34), and these worsening health-related lifestyle factors were related to T2DM (35–37).

The potential biological mechanisms that underlie the association of job strain with T2DM risk are complex. Neuroendocrine disorders may be the most key mechanism, including activation of sympathetic nervous system and dysregulation of the hypothalamic–pituitary–adrenal axis, which has been described in details in previous studies (38–40). Besides, except for contributing to worsening health-related lifestyle factors, job stress could also affect depressive symptoms (41) that was well-documented risk factor for T2DM (42), which may also be an important indirect mechanism.

The present meta-analysis has several strengths. We included only prospective cohort studies with a mean quality score of 7.8, which could ensure the high quality of our study. Besides, the exposure was defined clearly in our meta-analysis, and all included papers defined job strain according to JCQ or derivatives of the JCQ and scores of the validated JCQ based on the job strain model. Binary job strain and quadrants based on the job strain model were analyzed separately, which could make the results more reliable and accurate.

There were several limitations in our meta-analysis. Firstly, we focused on job strain, which is the most widely studied form of work-related stress. However, other work-related stress, such as effort–reward imbalance (43), job insecurity (44) as well as various sources of stress outside work (45) were not considered in our study. Thus, our findings are likely to underestimate the overall impact of work-related stress on T2DM risk. Secondly, ascertainment of T2DM varied between these studies, and case ascertainment in some studies was based on self-reports. Thus, there could have been misclassification of T2DM. Last but not least, critics have commented that the crude median split definition of job strain leads to underestimation of the true magnitude of the association (46, 47), as most participants in an epidemiological study are likely to center round the middle.

### Concluding remarks

In summary, our analysis indicates that job strain may increase T2DM risk, especially among women. Prospective cohort studies with larger sample sizes and longer follow-up times are warranted to probe the potential mechanisms and establish causality.

### Conflicts of interest

The authors declare no conflicts of interest.

### Funding

The study was supported by National Natural Science Foundation of China (81903291, (81703203), the Fundamental Research Funds for the Central Universities (2019kfyXJJS032), Hubei Province health and family planning scientific research project (WJ2017M203), and Wuhan municipal health Commission (WG16B08). The funding sources had no role in the design and conduct of the study; collection, management, analysis, and interpretation of the data; preparation, review, or approval of the manuscript; and decision to submit the manuscript for publication.

## Supplementary material

Supplementary material
