# Supplementary material for: Is job strain associated with a higher risk of type 2 diabetes mellitus? A systematic review and meta-analysis of prospective cohort studies
Source: Scand J Work Environ Health. 2021 Apr 27;47(4):249–57. doi: 10.5271/sjweh.3938 (PMC8091067; doi:10.5271/sjweh.3938)
Supplement: Supplementary material [file SJWEH-47-249-S001.pdf]

Is job strain associated with a higher risk of type 2 diabetes mellitus? A systematic review and meta-analysis of prospective cohort studies[1]

by Wenzhen Li, MD, Guilin Yi, MS, Zhenlong Chen, MS, Xiayun Dai, MD, Jie Wu, MS, Ying Peng, MS, Wenyu Ruan, BS, Zuxun Lu, MD, Dongming Wang, MD[2]

1. *Supplementary material*
2. *Correspondence to: Dongming Wang, Department of Occupational & Environmental Health, School of Public Health, Tongji Medical College, Huazhong University of Science and Technology, Wuhan, Hubei 430030, China. [E-mail: 648283002@qq.com]*

Table S1 Quality assessment of included studies using the Newcastle-Ottawa Scale

Study (year of publication)	Selection process of cohorts	Comparability of cohorts	Identification of the exposure and the outcomes of study participants	total score
Miriam Mutambudzi(2016)	3	2	2	7
Cornelia Huth(2014)	4	2	3	9
Solja T. Nyberg(2014)	4	1	2	7
Norito Kawakami(1999)	3	1	3	7
Alexandros Heraclides(2009)	3	2	3	8
Candyce H. Kroenke(2006)	3	2	3	8
M. Norberg(2007)	4	2	2	8
Anna-Karin Eriksson(2013)	4	2	3	9
K.-Y. Pan(2017)	3	2	2	7
Chie Kaneto(2013)	4	2	1	7

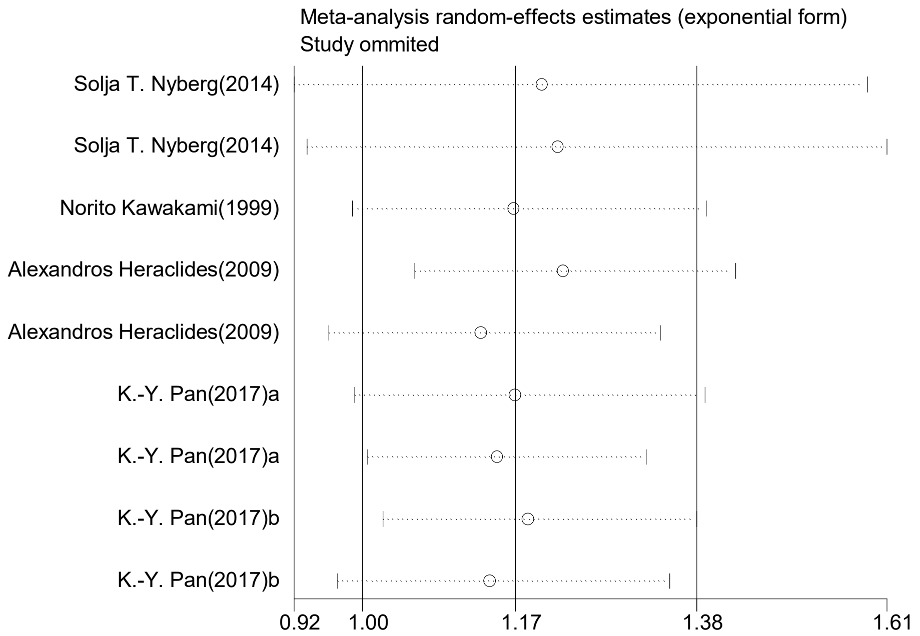

Figure S1(A) Sensitivity analyses for studies of high job strain withT2DM risk

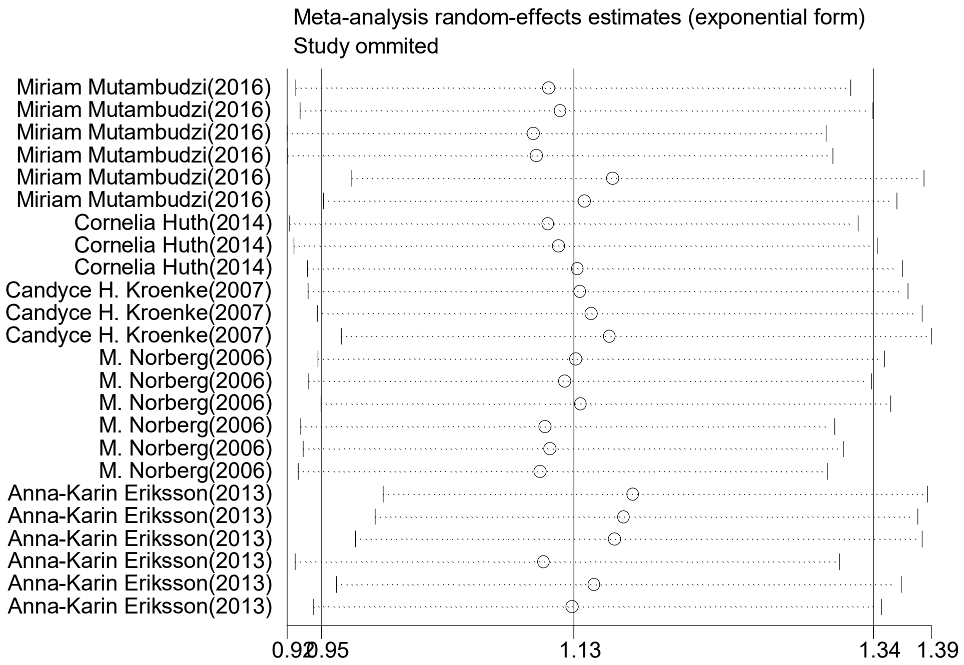

Figure S1(B) Sensitivity analyses for studies of job strain model quadrants withT2DM risk

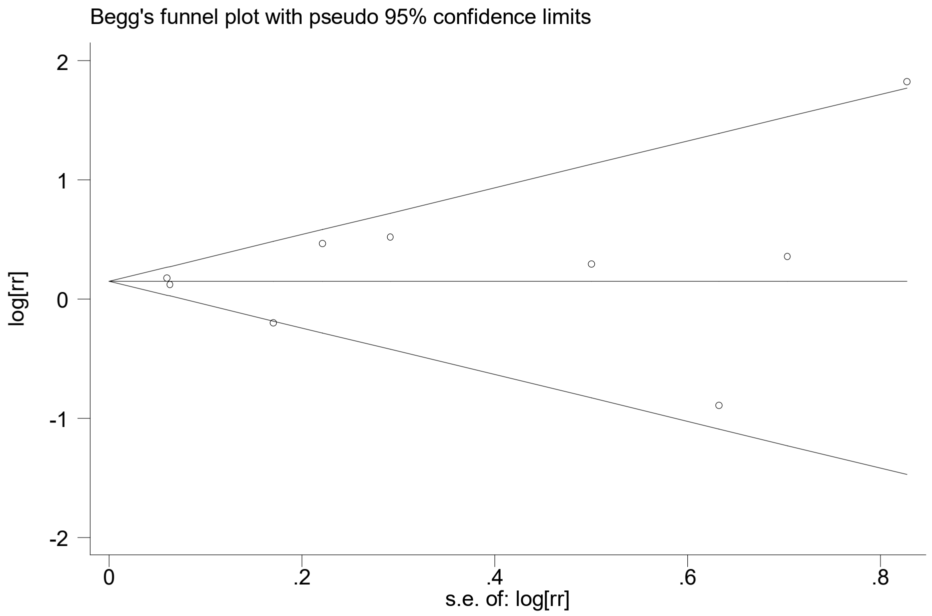

Figure S2(A) Funnel plot for studies of high job strain in relation to T2DM risk

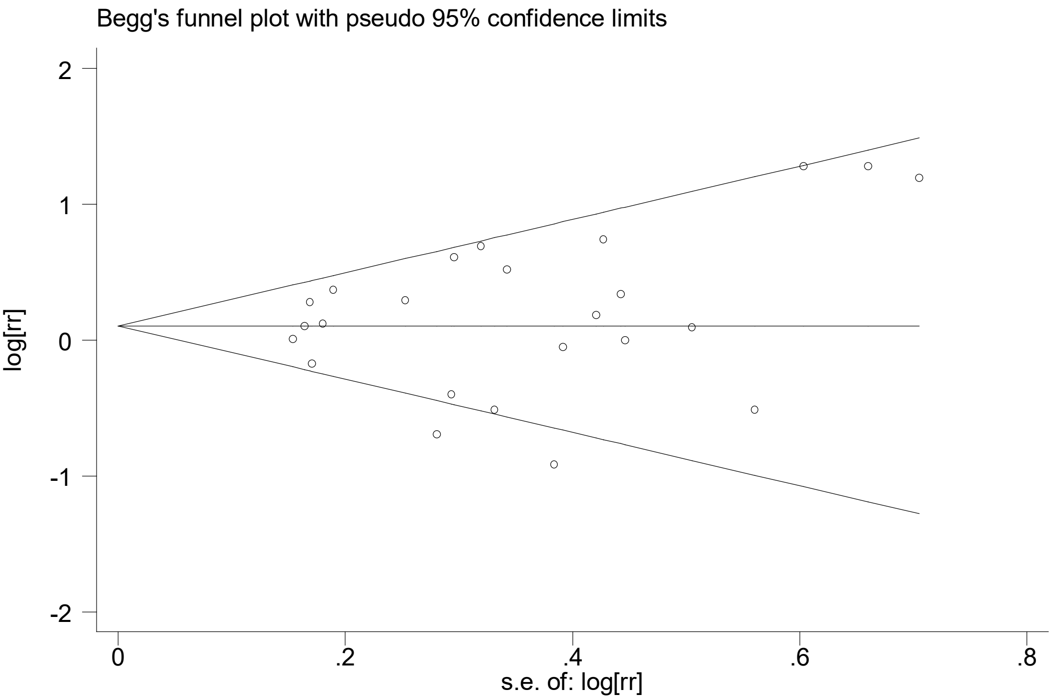

Figure S2(B) Funnel plot for studies of job strain model quadrants in relation to T2DM risk
